# Perturbation of 3D nuclear architecture, epigenomic dysregulation and aging, and cannabinoid synaptopathy reconfigures conceptualization of cannabinoid pathophysiology: part 1–aging and epigenomics

**DOI:** 10.3389/fpsyt.2023.1182535

**Published:** 2023-09-05

**Authors:** Albert Stuart Reece, Gary Kenneth Hulse

**Affiliations:** ^1^Division of Psychiatry, University of Western Australia, Crawley, WA, Australia; ^2^School of Medical and Health Sciences, Edith Cowan University, Joondalup, WA, Australia

**Keywords:** cannabis, cannabinoid, genotoxicity, epigenotoxicity, transgenerational inheritance

## Abstract

Much recent attention has been directed toward the spatial organization of the cell nucleus and the manner in which three-dimensional topologically associated domains and transcription factories are epigenetically coordinated to precisely bring enhancers into close proximity with promoters to control gene expression. Twenty lines of evidence robustly implicate cannabinoid exposure with accelerated organismal and cellular aging. Aging has recently been shown to be caused by increased DNA breaks. These breaks rearrange and maldistribute the epigenomic machinery to weaken and reverse cellular differentiation, cause genome-wide DNA demethylation, reduce gene transcription, and lead to the inhibition of developmental pathways, which contribute to the progressive loss of function and chronic immune stimulation that characterize cellular aging. Both cell lineage-defining superenhancers and the superanchors that control them are weakened. Cannabis exposure phenocopies the elements of this process and reproduces DNA and chromatin breakages, reduces the DNA, RNA protein and histone synthesis, interferes with the epigenomic machinery controlling both DNA and histone modifications, induces general DNA hypomethylation, and epigenomically disrupts both the critical boundary elements and the cohesin motors that create chromatin loops. This pattern of widespread interference with developmental programs and relative cellular dedifferentiation (which is pro-oncogenic) is reinforced by cannabinoid impairment of intermediate metabolism (which locks in the stem cell-like hyper-replicative state) and cannabinoid immune stimulation (which perpetuates and increases aging and senescence programs, DNA damage, DNA hypomethylation, genomic instability, and oncogenesis), which together account for the diverse pattern of teratologic and carcinogenic outcomes reported in recent large epidemiologic studies in Europe, the USA, and elsewhere. It also accounts for the prominent aging phenotype observed clinically in long-term cannabis use disorder and the 20 characteristics of aging that it manifests. Increasing daily cannabis use, increasing use in pregnancy, and exponential dose-response effects heighten the epidemiologic and clinical urgency of these findings. Together, these findings indicate that cannabinoid genotoxicity and epigenotoxicity are prominent features of cannabis dependence and strongly indicate coordinated multiomics investigations of cannabinoid genome-epigenome-transcriptome-metabolome, chromatin conformation, and 3D nuclear architecture. Considering the well-established exponential dose-response relationships, the diversity of cannabinoids, and the multigenerational nature of the implications, great caution is warranted in community cannabinoid penetration.

## 1. Introduction

From recent investigations, four important overarching themes have emerged, which assist and direct an updated understanding of cannabinoid pathophysiology. In particular, the integrated multi-channel study of the genome, epigenome, transcriptome, proteome, metabolome, and numerous histone modifications has provided unprecedented insights into the control of gene transcription and cellular behavior both in normal growth and development and in numerous diseases (1).

First, by introducing DNA breaks (2–8) and inducing global DNA hypomethylation (9–13), cannabis directly drives cellular and organismal aging, including epigenomic DNA methylation age (14), increases cardiovascular–organismal age (15), and results in the increased incidence of acute and chronic physical and mental diseases, including protean psychiatric disorders (16).

Second, by disrupting the basic epigenomic machinery of DNA methylation (9–13, 17–20), as well as histone methylation and acetylation (21, 22), the coordination between the histone code and the methylome (11), and the machinery for nucleosome repositioning (11), cannabinoids change the way the cell nucleus processes information, including gene transcription. This includes perturbation of the chromatin looping structures from which genes are transcribed by altering the CTCF (CCCTC-binding factor) boundary elements that delimit and define the loops and by disrupting the cohesin motors that drive and form DNA loops (11). Together, these changes significantly alter the nuclear structure and enhancer–promoter interactions at an ultrafine resolution and can thereby prime cells for malignant transformation. Such actions on germ cells (eggs and sperm) lead to congenital anomalies and conceptus aging.

Third, there is growing evidence that multiomics interactions between the metabolome, the microbiome, the immunome, the genome, and the epigenome are all interdependent and interrelated and cannot be properly understood without reference to one another. As cannabis is known to disrupt each level of this intercalated cascade, this must be borne in mind when considering its plethoric presentations. For example, it is known that the metabolome controls the epigenome in many ways (23, 24) and can reset the lineage determination set point of the cell away from full differentiation and toward dedifferentiation and premalignant preparedness for transformation (the Warburg effect) (25). Cannabis disrupts the post-translational tubulin code at several points (11, 26), inducing chromosomal missegregation, micronucleus formation (27–32), cell transformation, neurodevelopmental and congenital defects, and fetal loss (27, 33–37). The microbiome signals via the metabolome and the immunome (38–40). Similarly, the glycome bidirectionally interacts with the metabolome and the remaining cellular machinery and modulates the epigenome, the immunome, the microbiome, and aging (41–52).

Mitochondria are a major signaling hub within the cell (23, 24). The well-known inhibitory activities of cannabinoids on many mitochondrial functions (53–66) imply mitonuclear stress signaling to the nucleus (23, 24), endoplasmic stress response induction (67–72), and reduced supply of energy and metabolomic substrates to the epigenomic machinery (23, 24). Mitochondrial inhibition also increases cellular lactate (73, 74), which, in turn, increases the lactylation of major rate-limiting enzymes in glycolysis, oxidative phosphorylation, and related pathways (73, 74), alters the epigenomic structure and again dedifferentiates the cell, and primes it toward malignant pretransformation (73, 74). Increased shunting of glucose through the pentose phosphate pathway changes the synthesis of glycan groups for local and intercellular use (75) and can thus change the local tissue and tumor microenvironment.

Fourthly, the physiologic function of endocannabinoids under normal conditions is to signal the end of a synaptic trafficking event retrogradely from the post-synaptic membrane to the pre-synaptic membrane (76–78). It is well established that synapse formation and growth are activity-dependent and that much of the computation occurs based on the structure, size, strength, and chemical nature of the synapse (79–87), that is, the locus of many computations, including memory, is primarily synaptic (79–87). Flooding the synapse with xenophytocannabinoids, especially chronically, clearly grossly disrupts and perturbs this delicate process, thereby deranging the basic unit of brain computation (76, 88–90) and inducing downregulation of CB1Rs^1^ in the ventral striatum of the midbrain (91, 92). This cannabinoid synaptopathy is exacerbated by the usually pro-inflammatory actions of many cannabinoids on brain astrocytes and microglia (93–102), as well as the powerful negative effect of cannabinoids on oligodendroglial progenitor cells (OPCs). These cells create the myelin sheaths that nourish and preserve axons and white matter tracts. Additionally, the negative effects of cannabinoids on brain neuronogenesis (103–106) contribute to the exacerbation. These effects have been found to accumulate and cause a high degree of cortical white matter disconnection in chronic cannabis users (107).

### 1.1. Cannabinoid signaling

The complexity of the endocannabinoid system (ECS) in terms of its two main endogenous ligands and their synthesizing and metabolizing enzymes, as well as the many lipid molecules that interact with the ECS, has been described by many authors. However, for present purposes, it is important to appreciate that cannabinoid signals are neither simple nor binary, nor do they occur only at one locus. Endogenous cannabinoid receptors include but are not limited to the following: CB1R and CB2R^2^, vanilloid receptors TRPA1, TRPV1, TRPV4, and TRPV5^3^ (108, 109), nuclear receptors PPARα and PPARγ^4^, and GPCR119, GPR18 and GPR55^5^ receptors (110–112), the smoothened receptor in the sonic hedgehog pathway (11, 108, 111, 113–119), NMDAR^6^ (109), GABA_A_Rs,^7^ glycine receptors, 5HT_3_Rs^8^ (120), adenosine receptors (121), voltage-dependent anion channels (VDAC1–L-, N-, and P/Q-type calcium channels) (109, 122), and potentially others (112, 123). CB1Rs are located on the mitochondrial outer membrane and in the endoplasmic reticulum (66, 124–127). Between the inner and outer mitochondrial membranes and the intermembrane space, mitochondria possess all of the signaling machinery of the endocannabinoid system (66, 124–127). PPAR receptors are located in the cell nucleus (128–135). CB1R and CB2R activation leads to increased calcium fluxes into cells, increased potassium efflux from cells, and reduced cellular cAMP levels, which lead to the retrograde suppression of activity in the excitatory and inhibitory pre-synaptic nerve terminal (121).

Cannabinoid receptors have been observed to heterodimerize with opioid, dopamine, adrenergic, adenosine, serotonin, and angiotensin receptor type 2, as well as sonic hedgehog receptors (76, 77, 89–91, 108, 121, 136). CB1Rs also heterodimerize with tyrosine kinase receptors of the neurotrophin and epidermal growth factor receptor classes, among others (121). Indeed, heterodimerization between CB1R and CB2R has also been identified (121, 137). In most cases, the activities of these heterodimeric receptors are not well studied (121).

In this first paper, we aim to set out a narrative conceptual review of how and why gene expression is altered in cannabis use disorder and in the manner in which this disordered chromatin architecture is likely to underlie the findings of modern epidemiologic studies of cannabinoid teratogenesis and carcinogenesis in large nationwide and continental populations. Therefore, our perspective is both gene-centric and focused on a chromatin conformation-based analysis. Therefore, we first consider aging and then move on to epigenomics and the many ways in which these two major areas interact with each other.

## 2. Aging–epigenomic aging

### 2.1. Twenty stigmata of aging in cannabis dependence

Fifteen hallmarks of aging have been described in cannabis dependence, including (1) increased acute and chronic physical and mental illness (138), (2) acceleration of cardiovascular and organismal age (15), (3) endocrine disruption, particularly of the hypothalamo-pituitary-gonadal axis (139, 140), (4) mitochondrial inhibition (141–144), (5) DNA hypomethylation and advanced epigenetic age (14, 145–147), (6) neuroinflammation accompanying cannabis-associated mental illnesses (148–173), (7) cirrhosis (174–176), (8) degeneration of oocytes and sperm (177, 178), (9) increased carcinogenesis (28, 179–190), (10) heightened rates of many congenital anomalies and teratologic syndromes (27–29, 191–207), (11) telomerase inhibition (11, 208), (12) chromosomal damage (2, 4, 8, 178), (13) reduction in histones (5, 21, 26, 209–211), (14) immunostimulation (93, 94, 99–101, 212–217), and (15) elevated mortality rates in long-term users (218–229). These are elaborated in detail elsewhere (31, 185, 230).

To this list, an additional five features of aging that are also characteristic of cannabis dependence can be added. These include (16) a reduced respiratory exchange ratio (the amount of CO2 produced for oxygen taken up by tissues and organisms and clearly reduced due to the well-characterized inhibition of mitochondrial metabolism), (17) reduced ATP production by mitochondria, (18) increased extra-chromosomal DNA circles, (19) an increase in double-stranded DNA breaks, and (20) a reduction in lamin B (22). Double-stranded DNA breaks are a severe threat to cell survival, and the ability of cells to closely control their occurrence correlates well with organismal lifespan (22). Lamin B is a component of the internal nuclear envelope that functions to assist with gene silencing. Its reduction has been linked with increased senescence-associated β-galactosidase positive cell staining and an increase in the release of pro-inflammatory interleukin-6, CCL2^9^, CCL20^10^, and LINE1^11^ retrotransposons and inhibitors of apoptosis (IAP).

Therefore, these 20 features of aging together constitute strong and robust evidence for the acceleration of cellular aging by cannabis, similar to the evidence observed with tobacco use (22, 231). This implies that studies in aging have a direct relevance to understanding the effects of various cannabinoids on cell behavior.

### 2.2. Review of a key aging pathophysiology study

A team of 64 investigators from Harvard Medical School and their collaborators used an “Inducible Changes to the Epigenome” (ICE) protocol in mouse cells to show that the induction of only 20 double-stranded DNA breaks (DSBs) causes the epigenomic machinery on the genome to regenerate. This regeneration occurs in such a manner that the overall level of cell definition in the Waddington epigenetic landscape and the level of DNA methylation are reduced, while the epigenomic age is advanced. In the study, the rearrangement of the epigenomic apparatus was presumed to cause the redistribution of the DNA methylation machinery, which underlay the reduction in DNA methylation (22). The study showed that both gene activating (H3K27ac^12^ and H3K4me3^13^) and gene repressive (H3K9me3^14^ and H3K27me3^15^) epigenetic marks were downregulated by DSBs so that the normal epigenomic definitions between cells were blurred.

Concomitantly, the boundary function that controls chromatin loop formation and gene expression and directs enhancer–promoter interactions was reduced so that the enhancer function became misdirected aberrantly toward anomalous promoters. In general, cells became less well differentiated. Several examples of anomalous cellular dedifferentiation were documented, including fibroblasts that expressed neuronal genes, muscle cells that expressed spleen and immune genes, including major histocompatibility class II genes, and muscle cells that increased epithelial–mesenchymal transition in renal glomerular parietal lining epithelial cells (22).

Genes expressed in development, such as HoxA and Wnt genes, were found to be specific targets of this epigenomic de-programming. This was believed to be because they were poised for activation to assist with tissue repair in the event of some local injury or insult. The proteins coded by these genes are part of the DSB repair machinery; therefore, they were recruited to the DNA break sites together with other complexes (22).

Many aspects of aging were accelerated in ICE mice, including reduced short- and long-term memory and reduced physical coordination when walking, reduced body weight, reduced mobility at night, reduced fat mass, reduced strength, reduced hearing, cataract formation, reduced glomerular size, reduced skeletal mass, shorter running time and distance, reduced muscle ATP, mitochondrial DNA, and muscle lactate, hair graying and thinner skin, and increased brain neuroinflammation, including 1.6x more activated astrocytes and 3.5 times more activated microglia. The epigenetic age of the blood and skeletal muscle was 50% advanced in ICE mice (22).

The expression of the classic senescence gene CDKN1A^16^ (encoding P21) was upregulated. The expression of the canonical epigenomic activators of gene expression H3K27ac and H3K56ac was downregulated. The expression of H3K27ac was inversely correlated with its baseline expression, implying that it was most reduced at promoters where it was previously highly expressed and vice versa. Since H3K27ac, the classic epigenomic signal for gene activation, is most enriched at the tissue- and cell lineage-defining superenhancers, these were the epigenomic loci most weakened by these rearrangements.

Significantly, 50% of the top 20 programs identified by a Gene Ontology search were involved in developmental and organ patterning processes (22). Organ systems that were inhibited by the ICE treatment included the adult and fetal brain, heart, lungs, gastrointestinal organs, and muscle cells. Gene Ontology terms that were suppressed included the following: regulation of blood coagulation, regulation of transmembrane receptor serine/threonine kinase pathways, negative regulation of endothelial cell proliferation, regulation of coagulation, skeletal system morphogenesis, single organism signaling, pattern specification processes, bone morphogenesis, tissue development, skeletal system development, organ development, transcription from RNA polymerase II promoter, cell communication, odontogenesis, negative regulation of cell adhesion, specification of organ identity, bone development, regulation of wound healing, regulation of smoothened signaling pathway (sonic hedgehog), and negative regulation of cell proliferation. It is clear from this extensive list that many key developmental processes were extensively suppressed.

Along with the weakening of superenhancers, superanchors were also weakened. This was demonstrated by showing that aberrant enhancer–promoter interactions occurred when the three-dimensional chromatin looping was assessed (22). Therefore, these findings together revealed that cellular identity was weakened and indeed disrupted.

Importantly, a highly broad and diverse spectrum of immune gene superenhancers exhibited an increase in H3K27ac in many cell types, while the transcriptional programs for other organ genes–such as heart, brain, livers, kidneys and muscle cells–were suppressed. Immune pathways that were increased by the application of the Gene Ontology analysis included cell activation, leukocyte activation, lymphocyte activation, T-cell activation, regulation of T-cell activation, regulation of lymphocyte activation, regulation of leukocyte activation, antigen processing, regulation of immune cell processes, lymphocyte differentiation, T-cell differentiation, peptide antigen processing via MHC, regulation of lymphocyte proliferation, positive regulation of lymphocyte activation, hemopoietic/lymphoid organ development, regulation of mononuclear cell proliferation, hemopoiesis, and leukocyte cell adhesion (22). The activity of the H3K27ac signal in immune superenhancers in the spleen was approximately double that of controls. This list demonstrates the profound extent of pro-inflammatory, pro-immune reprogramming created by the induced pro-aging genomic–epigenomic damage.

Epigenomic factors known to be involved in DSB repair included SIRT1^17^, SIRT6, HDAC1^18^, and PARP1^19^. It was shown that they relocalized from the genome to the sites of DSBs. This mobilization of epigenomic silencers, in turn, induced the mobilization of retrotransposons and mobile elements of the genome, both of which lead to genomic instability and potently stimulate innate immune pathways (22).

Importantly, they also showed that increased epigenetic age was linked to an increase in DSBs. Thus, DSBs were shown to drive epigenomic age, and epigenomic age was shown to drive DSBs, forming a positive feedback loop.

Importantly, all of these adverse changes could be reversed by using three of the Yamanaka stem cell factors Oct3/4, Sox2, and Klf4 (OSK), thereby demonstrating that aging could be modulated both forward and backward by manipulating the genome (through DSBs) and epigenome (22). When the OSK regenerative factors were administered by intravitreal injection into the eyeball, there was a marked regeneration of the retinal ganglion cells, which in older mice are normally highly degenerative. This phenotype was replicated in ICE mice. Gene Ontology pathways that were enriched in these optic nerves and retinae included nervous system development, system development, neurogenesis, generation of neurons, multicellular organism development, regulation of multicellular processes, development of anatomic structures, developmental processes, regulation of localization, regulation of biologic quality, regulation of transsynaptic signaling, modulation of chemical synaptic signaling, regulation of ion transport, neuronal differentiation, response to external stimuli, neuronal development, regulation of transport, multicellular organismal processes, synaptic signaling, and cellular development processes (22). Thus, many key neural regenerative pathways were strongly restored by OSK therapy.

Therefore, these workers could ascribe the aging process itself to a loss of epigenomic information, which was bidirectionally coordinated with related processes such as genomic breaks, immune stimulation, and stem cell impairment, as well as developmental and regenerative programs.

### 2.3. Relevance to cannabinoid pathophysiology

As indicated above, these epigenomic and functional studies of aging are directly relevant to patients exposed to cannabis for many reasons. As the authors state, there is no question that such findings apply to tobacco exposure (22), and since cannabis has currently been shown to be a more potent genotoxin than tobacco in multiple studies (189, 191, 202, 232), these observations apply even more so to cannabinoids.

It is important to note how closely cannabis phenocopies this described process. DSBs (2–8), DNA hypomethylation (9–13, 233), and weakened CTCF boundary elements (11), which are the core components of the above schema, are all well described following cannabis exposure (11).

The involvement of key developmental processes Wnt, HoxA, and sonic hedgehog in the above results explains for stroke the implication of cannabinoids in a wide variety of teratogenic, developmental, and neurodevelopmental congenital anomalies, as documented in Colorado, Hawaii, the USA, Canada, Australia, and Europe (27–29, 191–205, 207). This description fits well with the wide variety of congenital anomalies that have been linked with cannabis, including those of the cardiovascular, central nervous, gastrointestinal, chromosomal, limb, uronephrological, body wall, and orofacial systems, as well as in the general embryo (27–29, 191–205). Congenital anomalies that have been linked to cannabis exposure in the USA were anophthalmia/microphthalmia, anotia/microtia, aortic valve stenosis, atrial septal defect, biliary atresia, bladder extrophy, choanal atresia, cleft palate alone, cleft lip alone, cleft lip with cleft palate, cleft lip with or without cleft palate, cloacal extrophy, club foot, coarctation of the aorta, common truncus, congenital cataract, congenital dislocation of the hip, congenital posterior urethral valve, deletion of 22q11.2, diaphragmatic hernia, Ebstein's anomaly, encephalocele, epispadias, esophageal atresia with or without tracheesophageal atresia, Hirschsprung's disease, congenital megacolon, hydrocephalus without spina bifida, hypospadias, interrupted aortic arch, microcephalus, obstructive genitourinary defect, omphalocele, patent ductus arteriosus, pulmonary valve atresia, pulmonary valve atresia and stenosis, rectal and large intestinal atresia and stenosis, reduction deformity upper limbs, reduction deformity lower limbs, renal agenesis and hypoplasia, small intestinal atresia/stenosis, trisomy 13, trisomy 18, trisomy 21 (Down's syndrome), Turner's syndrome, and ventricular septal defect (192, 202, 205).

The unequivocal demonstration that cellular dedifferentiation occurs due to DNA demethylation, weakening of superenhancers and superanchors, aberrant promoter–enhancer communication, and retrotransposon activation clearly explains why many diverse tissues are primed by cannabis for malignant transformation, which addresses the issue of why so many cancers have been epidemiologically linked with cannabis (25, 28, 32, 179–188, 220, 234–247). Cancers that were linked with cannabis exposure in Europe were all cancers, excluding non-melanoma skin cancer, bladder, brain, breast, colorectal, Hodgkin's, kidney, larynx, liver, lung, melanoma, multiple myeloma, myeloid and lymphoid leukemias, non-Hodgkin's lymphoma, and esophagus, oropharynx, ovary, pancreas, prostate, stomach, testis, thyroid, and uterine cervix cancers (189).

For many of these tumors, positive dose-response effects have been described (220, 238, 240, 241). There are also many examples of inheritable tumors due to the intergenerational transmission of major genotoxic lesions (248, 249), including acute lymphoid and myeloid leukemias, rhabdomyosarcoma, and neuroblastoma (28, 186, 188, 250–252).

Importantly, cannabis has been shown to be a driver of rising rates of breast, testicular, liver and pancreatic cancers in adults (28, 183, 184, 187, 190, 234, 253–255) and of total pediatric cancer (188) and acute lymphoid leukemia (188) in children. Most of the studies referred to in this paragraph were conducted in space–time contexts and in causal inferential paradigms to allow for the formal quantitative investigation of epidemiologically causal pathways to be investigated.

Indeed, a question has been formally posed (190, 234) regarding whether cannabis might be a major factor underlying the modern resurgence of several types of cancer developing in patients younger than 50 years (235).

The close, reciprocal, and mutually reinforcing relationship between the DSB-inducing actions of cannabinoids and epigenomic dysregulation is also clarified. Moreover, the manner in which the classically described DSB induction and chromosomal clastogenicity are linked to the newly defined epigenomic dysregulation is also explicated.

Multiple cannabinoids are known to impede mitochondrial and intermediate metabolism (55, 56, 65, 66, 122, 256–263). This necessarily reduces the availability of methyl and acetyl groups for methylation and acetylation reactions, which, by definition, reduces both the epigenomic instructions written to the DNA and gene availability and, thereby, “flattens” the epigenomic landscape [related Waddington's epigenomic valleys (264)].

Furthermore, DSB induction and various levels of epigenomic dysregulation also clarify not only the occurrence of cannabinoid-induced aging but also some of its likely cellular mechanisms.

With this argument established on theoretical grounds, all of these features require verification in the cellular models of cannabinoid cytotoxicity, genotoxicity, epigenotoxicity, and aging.

## 3. Epigenomics

### 3.1. Enhancer–promoter interactions

The human genome has approximately 25,000 genes and 1,000,000 enhancers (265). There is significant enthusiasm within the scientific community due to the development of low input chromosome conformation capture techniques for interrogating three-dimensional genome architecture within the nucleus, which allows for a detailed description of the manner in which genes are transcribed from chromatin loops that are formed when cohesin motors extrude DNA loops through their lumen (266). The cohesin complex is known to form loops around chromatin during chromosomal pairing, which occurs at the mitotic metaphase and also during gene transcription (267). These looping structures are constrained by boundary elements, which is most often CTCF^20^ (266–270) being the most common element. These boundary elements divide the chromatin into topologically defined domains for transcription (269). The minichromosome maintenance (MCM) complex has also been shown to block cohesin loop extrusion and act as a boundary element (271). These domains are carefully constrained to usually contain both the gene promoter and the enhancers acting in *cis* (on the same chromosome), albeit some enhancers act at large distances over one megabase or on different chromosomes (in *trans*). Importantly, DNA methylation prevents the binding of CTCF to chromatin (272). These topologically defined domains are organized and clustered together inside the three-dimensional space of the nucleus into transcription factories. At present, this looping model has been demonstrated in many different tissues in both physiologic and pathologic states, including during embryonic development (273–276), during chondrogenesis (277), in normal tissues (278, 279), in the heart (280–282), in the brain (283–290), in T-cell differentiation (269, 291), for stem cells (292) during cellular reprogramming and dedifferentiation (22, 293, 294), and within many cancers (269, 291, 295–302). Thus, these looping structures bring together both the promoter and enhancers, usually within 300 nm, to control gene transcription. Indeed, it has been reported that 90% of the risk genes identified in genome-wide studies are located within non-coding genomic regions, especially in enhancers (265). Experimental and biostatistical studies have shown that clusters of enhancers work together synergistically and combinatorially (265, 270, 278).

Superenhancers are large groups of enhancers that are clustered on the genome and control the state of differentiation and cell lineage determination (267, 303–305). In other words, they are believed to determine whether a heart cell is a heart cell as opposed to a neuron or blood cell, for example. Superenhancers are extremely powerful and perform activities that are several orders of magnitude above ordinary enhancers; they may act either from the same chromosome or from another chromosome. The limits of superenhancers are protected by “superanchors,” which normally control their activity and reach (269). Clearly, their significant power confers great risk if their ability to stimulate transcription is misdirected, as indeed occurs in many cancers (267, 269, 300, 303, 304). These phenomena are referred to as “enhancer hijacking” and “silencer hijacking” (267). DNA hypomethylation caused the loss of CTCF boundary elements, resulting in the formation of neoloops even between adjacent chromosomes and leukaemogenesis through a gain of function related to this enhancer hijacking (267, 269, 306). Contrarily, the superenhancer dependence of many tumors becomes a particular vulnerability for therapeutic exploitation, and this is presently being intensively explored (307).

A crucial detailed longitudinal study of the changes in human and rat sperm induced by cannabis exposure and resolving after a period of cannabis abstinence has been published (11). Cannabis-dependent human volunteers and rats were exposed to cannabis and then underwent 11 weeks of documented abstinence from cannabis. Eleven weeks is the period one sperm cycle takes in humans. Epigenomic changes were then documented from a control state and longitudinally against earlier time points.

Since the control of enhancer–promoter looping interactions by boundary elements has currently become the basic model for controlling gene transcription, the observation in the Schrott dataset (11) that cannabis withdrawal disrupts the expression of CTCF carries profound implications, since CTCF is the basis of structure and order in the whole architecture of enhancer–promoter interactions. In the absence of proper CTCF boundary function, enhancers and promoters will inevitably be brought into inappropriate contact with severe sequelae, including disordered neurodevelopmental outcomes (269, 279, 283, 284, 287, 289, 308, 309) and many cancers (269, 295–302). Cancer can occur when a promoter region is inappropriately exposed to an enhancer region, thereby providing an inappropriate stimulus to gene transcription. Indeed, one powerful scenario is when a tissue defining superenhancer is brought adjacent to a strong oncogene, such as Myc or Notch, which can cause run away growth stimulation, which is a not uncommon scenario both in many leukaemias and solid tumours (265, 267, 269, 291, 296, 298–300, 310–312).

The main proteins comprising the cohesin ring may be listed as SMC1^21^, SMC3, RAD51^22^, and STAG^23^ proteins. Cohesin is involved in post-replicative DNA repair and transcriptional regulation, and it also plays an important role in pairing chromosomes (313). Therefore, the finding that there were 96 DMRs in the Schrott dataset for the structural maintenance of chromosomes (SMC) genes, 9 DMRs for RAD51, and 152 DMRs for the STAG proteins, comprising 257 hits, is crucial (11). Indeed, the significance of RAD51 epigenomic inhibition is amplified by its primary role as a key enzyme in the high-fidelity DNA repair pathway known as homologous recombination. When RAD51 expression is disabled, alternative lower fidelity error-prone DNA repair processes, such as mismatch repair (in stem cells) or theta end joining (in oocytes and in many cells) (272), are employed, and these lower fidelity pathways are inherently mutagenic. Importantly, sperm were shown to be particularly susceptible to DNA damage owing to their largely unmethylated DNA state, their DNA compaction in protamine barrels that are six times more tightly compressed than normal, and the complete absence of DNA repair machinery (272). For this reason, 80% of congenital disorders diagnosed postnatally have been ascribed to paternal contribution (272).

For example, in acute lymphoid leukemia (ALL), which is the most common childhood cancer that represents inherited genotoxicity and has previously been linked with community cannabis exposure (188), it was shown that a key driving mutation occurs in the GATA3 ^24^ enhancer, which changes chromatin conformation and gene expression (300). GATA3 is a pioneer factor that recruits the SMARCA4 (SWI/SNF-related, matrix-associated, actin-dependent chromatin regulator, subfamily A, member 4) ^25^ complex to open up the genome and sets in train a GATA3/CRLF2 ^26^/JAK2 ^27^/STAT5 ^28^ signaling pathway to leukaemogenesis (300). SMARCAs perform energy-dependent repositioning of nucleosomes and increase the accessibility of genes to the transcription machinery. GATA activation induces a state switch in the nuclear synthetic compartments (B (silent) to A (active transcription) compartment switching) for many genes. GATA3 overexpression induced enhancer hijacking (300). GATA3 activation has also been identified in many other hematologic malignancies, such as the Reed–Sternberg cells in Hodgkin's disease (300). Interestingly, GATA3-binding sites were located near the Philadelphia-like chromosome break point. However, this study could not demonstrate a causal link related to this issue. Widespread B to A compartment switching was also identified in another study of acute lymphoid leukemia (299). Importantly, the rs3824662 risk variant in the GATA3 promoter is inheritable (300).

Of further importance, there were 127 hits for GATA in the Schrott epigenomic cannabis screen (11). There were over 28 DMRs for actin-related proteins in the Schrott dataset (11). Seven DMRs were identified for SMARCAs 1, 2, 4, and 5 (11). Since SMARCAs are both ATP- and actin-dependent, and since cannabinoids disrupt both actin production and ATP synthesis as well as SMARCAs themselves, it follows that nucleosomal positioning and gene transcription are necessarily disrupted. SMARCAs have also been shown to be of pivotal importance in enhancer-addicted prostate cancer (302).

Therefore, to observe that cannabis significantly disrupts both CTCF as the fundamental boundary element defining transcription regions and the machinery and motors that drive chromosomal loop extrusion and orchestrate gene transcription is to necessarily point to a major disruption of the fundamental process of gene transcription.

It should also be observed that normal genomic processes can induce DNA breaks, including DNA transcription and duplication, base excision repair, and active DNA demethylation (314, 315).

### 3.2. Epigenomic memory

It has also been shown that many cell types record histories of past exposures in the highly complex post-translational codes in their epigenome, especially their histone codes (145, 146). These codes form memories. They are also advantageous in that should an inflammatory or toxic insult recur, gene cassettes are often poised for rapid reactivation and usually have a modified response, which may be either potentiated in the case of an infective insult (145) or ameliorated in the case of pancreatitis (316–318).

### 3.3. Cannabinoid impacts on epigenomic machinery

The study of Schrott and colleagues (11) also described the manner in which cannabis dependence and withdrawal disrupt the basic machinery of epigenetic regulation, including DNA methylation writers and erasers (DNMT1/3^29^ and TETs^30^), histone methylation and acetylation writers and erasers (KMTs^31^, KDMs^32^, KATs^33^, HDACs^34^, and sirtuins^35^), stem cell regenerative transcription factors, key elements of the polycomb repressive machinery, major ATP-dependent factors that reposition nucleosomes and enable new genes to be transcribed (SMARCA2/4^36^), and coordinators of epigenetic processes, including DNA methylation and histone post-translational modifications (UHRF1^37^). UHRF1 is also a key regulator of cell growth. Growth inhibition explains some of the growth-inhibitory actions of cannabis, as described in studies involving babies' heads, brains, and hearts (191, 195, 196, 201, 203, 205, 207, 319–321).

From this analysis and concise review, it can be observed that cannabis broadly disrupts the fundamental epigenomic machinery and necessarily disrupts the basic machinery of gene transcription, thereby disrupting normal promoter–enhancer interactions. Deleterious effects on neurodevelopment, patterns of congenital anomalies, and cancerogenesis, including heritable cancerogenesis, should be the expected outcomes and are indeed also the observed outcomes.

Through the induction of genome-wide relative DNA methylation (9, 12, 13), single- and double-stranded DNAs and chromosomal breaks (2–8), inhibition of mitochondrial metabolism by diverse pathways (55, 56, 66, 256–260, 322), and within the context of widespread epigenomic disruption and interference with the basic gene looping mechanism of gene transcription, cannabis will necessarily reorganize nuclear pathophysiology. This reorganization can lead to genomic instability, numerous adverse congenital outcomes, including neurodevelopmental outcomes, and cellular aging, according to recent epigenomic pathophysiologic descriptions (11, 22, 294, 323, 324).

It is also of interest to consider the overlap between genes described in certain syndromes and those known to be epigenomically perturbed by cannabis use. Some of the largest gene databases in the existing literature have been intersected in this way with the epigenomic cannabis screen of Schrott and colleagues. This has produced the data shown in Table 1.

**Table 1:** Syndromic genes identified in the schrott cannabis epigenomic screen (11).

**N**	**Group**	**Disorder**	**Genes identified in the Schrott database**	**Genes implicated**	**% Genes implicated in Schrott**	**Reference**
1	Brain disorders	Schizophrenia	597	685	87.10%	Trubetskoy V. Nature 2022; 604(7906): 502–508
2	Congenital anomalies	Oocytic Zar1 activation	162	208	77.88%	Cheng S, Science, 2022; 378 (6617)
3	Congenital anomalies	Sperm	2,974	4,930	60.34%	Chen Y, Cell Res. 2018; 28: 879–896
4	Brain function	Purkinje cells–cerebellum	282	487	57.91%	Chen X., Science 605 (7911): 722–727
5	Brain development	Mid-fetal brain, Human, M2 motor cortex	106	189	56.08%	Shibata M, Nature, 2021; 598(7881): 483–488
6	Autism	Autism	600	1,095	54.80%	Sfari Database
7	Brain disorders	Autism	600	1,095	54.80%	Sfari Database
8	Cancer	Acute myeloid leukemia–differentiation genes	12	22	54.54%	Zeng A. Nat. Medicine 2022; 28:1212–1223
9	Brain development	Mid-fetal brain, human, frontal lobes	43	82	52.43%	Shibata M, Nature, 2021; 598(7881): 483–488
10	Aging	Ovarian aging–meta-analysis	2,212	4,378	50.50%	Ruth K, Nature 2021; 596(7872):393–397
11	Congenital anomalies	Congenital heart disease	1,169	2,320	50.40%	Hill M, Nature 2022; 608(7921): 181–191
12	Brain function	Brain astrocytes	26	66	39.39%	Burda J Nature 2022; 606(7914): 557–564
13	Brain function	Brain astrocytes	17	58	29.31%	Burda J Nature 2022; 606(7914): 557–564
14	Brain function	Brain astrocytes	36	106	33.96%	Burda J Nature 2022; 606(7914): 557–564
12	Cancer	Medulloblastoma, gene subset N=4	2	4	50.00%	Gershanov S. Front. Oncology 2021; 11:637482
13	Brain disorders	Alzheimer's disease	721	1,614	44.67%	Park J, Nat. Commun. 2019; 10(1): 3090–3101
14	Brain development	Dorsolateral prefrontal cortex	596	1,338	44.54%	Ma S., Science 2022; 377(6614): 1511–1524
15	Cancer	Acute myeloid leukemia–Lin7 cluster	3	7	42.85%	Zeng A. Nat. Medicine 2022; 28:1212–1223
16	Aging	Ovarian Aging–genes	124	290	42.74%	Ruth K, Nature 2021; 596(7872):393–397
17	Cancer	Acute myeloid leukemia–most commonly mutated	15	36	41.67%	Bottomley D Cancer Cell 2022; 40(8):850–864
18	Cancer	Pancancer–overall	873	2,181	40.02%	Chen R Cancer Cell 2022; 40(8): 865–878
19	Brain development	Mid-fetal brain, human, M1 motor cortex	2	5	40.00%	Shibata M, Nature, 2021; 598(7881): 483–488
20	Congenital anomalies	Preeclampsia	489	1,234	39.62%	Moufarrej M, Nature 2022; 602(7898): 689–694
21	Brain disorders	Alzheimer's disease	26	66	39.39%	Burda J., Nature 2022; 606(7914); 557–564
22	Cancer	Pancancer–low risk	377	967	38.98%	Chen R Cancer Cell 2022; 40(8): 865–878
23	Aging	Heterchronic parabiosis	8,216	21,176	38.79%	Ma S Cell Stem Cell 2022; 29:990–1005
24	Aging	Aging hemopoietic stem cells–genes	8,216	21,176	38.79%	Adelman E. Cancer Discover. 2019; 9(8):1080–1101
25	Cancer	Acute myeloid leukemia–overall	29	81	35.80%	Zeng A. Nat. Medicine 2022; 28:1212–1223
26	Cancer	Acute myeloid leukemia–druggable genes	289	810	35.67%	Bottomley D Cancer Cell 2022; 40(8):850–864
27	Aging	Aging hemopoietic stem cells–DMR's	526	1,499	35.09%	Adelman E. Cancer Discover. 2019; 9(8):1080–1101
28	Aging	Heterchronic parabiosis–HetO-IsoO	2,916	8,513	34.24%	Ma S Cell Stem Cell 2022; 29:990–1005
29	Brain disorders	Spinal cord injury	36	106	33.96%	Burda J., Nature 2022; 606(7914); 557–564
30	Brain function	Brain astrocytes	9,025	26,688	33.82%	Edno F, Science 2022; 378(66619): 514–525
31	Cancer	Medulloblastoma, gene subset N=12	4	12	33.33%	Gershanov S. Front. Oncology 2021; 11:637482
32	Cancer	Cancer driver genes (COSMIC)	5,260	15,827	33.23%	Sondhka Z Nature Rev Cancer 2018; 18:696–705
33	Brain development	Mid-fetal brain, human, prefrontal lobes	38	118	32.20%	Shibata M, Nature, 2021; 598(7881): 483–488
34	Cancer	Medulloblastoma	6,191	20,196	30.65%	Gershanov S. Front. Oncology 2021; 11:637482
35	Aging	Aging hemopoietic stem cells–DEG	340	1,133	30.00%	Adelman E. Cancer Discover. 2019; 9(8):1080–1101
36	Cancer	Pancancer–High Risk	496	1,214	29.42%	Chen R Cancer Cell 2022; 40(8): 865–878
37	Cancer	Acute myeloid leukemia	5	17	29.41%	Ng S, Nature 2016; 540(7633):433-−437
38	Brain disorders	Endotoxaemia (with LPS)	17	58	29.31%	Burda J., Nature 2022; 606(7914); 557–564
39	Cancer	Acute myeloid leukemia–overall	1,114	3,879	28.71%	Bottomley D Cancer Cell 2022; 40(8):850-864
40	Aging	Mouse aging	2,847	10,071	28.26%	Sleiman M Science 2022; 377(6614): 1508–1520
41	Cancer	Medulloblastoma, gene subset N=32	9	32	28.10%	Gershanov S. Front. Oncology 2021; 11:637482
42	Cancer	Acute myeloid leukemia–classifiers	28	100	28.00%	Zhang S J. Oncology 2022; 2022:7727424
43	Congenital anomalies	Oocytes	1,211	4,363	27.75%	Cheng S, Science, 2022; 378(6617)
44	Cancer	Medulloblastoma, gene subset N=22	6	22	27.20%	Gershanov S. Front. Oncology 2021; 11:637482
45	Aging	Heterchronic parabiosis–key genes	253	1,000	25.30%	Ma S Cell Stem Cell 2022; 29:990–1005
46	Cancer	Acute myeloid leukemia–DEG's	37	147	25.17%	Zhang S J. Oncology 2022; 2022:7727424

From Table 1, it can be observed that the overlap runs from 25.17% for acute myeloid leukemia and 25.3% in aging to 77.9% for congenital anomalies and 87.1% for schizophrenia. The autism screen is also of particular interest. The dataset used for the assessment was the Sfari database, which contains 1,095 genes and is the world's largest autism gene set database (325). The common intersected fraction identified with the Schrott epigenomic screen with the autism dataset was 54.8%.

### 3.4. Exponentiation

Substantial experimental evidence points toward the conclusion that the effects of cannabinoids are exponential and that it must be assumed that this is a normal class effect in the low micromolar range. This exponentiation applies to both its genotoxic (8, 113, 326–334) and metabolic effects (53–58). Since these epigenomics and metabolomics are closely related, this implies that this exponentiation is compounded in this case.

The low micromolar serum level is readily reached in patients who consume cannabis either regularly or daily (335). This issue is exacerbated by the accumulation of cannabinoids in tissues and their generally long tissue half-life (335).

The issue of exponential dose-response effects is of great importance in the public health context. When legislation exists, which attaches penalties to cannabis use, cannabis use is naturally discouraged. However, under decriminalized or legalized legislative frameworks, cannabis use has been shown many times to increase (336–341), along with an increase in the potency of the THC or cannabidiol products consumed. This rise is accompanied by the number of individuals who consume cannabis on a relatively intense or daily basis. Clearly, this places a significant number of people in the community into a high cannabis exposure zone relatively abruptly, where adverse genotoxic and neurotoxic outcomes become more commonplace.

For these reasons, it is envisaged that the triple confluence of rising cannabis prevalence rates, intensity of use rates, and cannabinoid potency will manifest relatively abruptly as steep rises in adverse mental health, as well as teratologic, carcinogenic, and age-related outcomes, as are indeed being observed and documented in several jurisdictions (16, 25, 27–32, 179–187, 189–205, 207, 232, 234–237, 321, 342–345).

#### 3.4.1. Fetal alcohol syndrome–fetal cannabinoid syndrome

The incidence of fetal alcohol syndrome (FAS) is increasing in many places. Indeed, a recent space–time and quantitative causal inference study in Europe showed that FAS was rising in association with increased cannabis use (201). This result went beyond merely reporting an association because it has currently been well established that FAS is mediated largely via the CB1R cannabinoid receptor (111, 114–117, 346–355), with GABAergic neurons shown to be particularly susceptible (114). This effect is also mediated by the sonic hedgehog receptor (shh), where cannabinoids bind to the shh-smoothened receptor (113, 114, 116, 118).

Indeed, a remarkably close phenotypic resemblance between infants exposed antenatally to cannabis and alcohol has been noted by many investigators (113–115).

Moreover, cannabis and alcohol compound the foetotoxic effects of each other so that their combined effect is potentiated (111, 113–115, 347, 351, 356). A corollary of this is that multisystem foetotoxic effects manifest at otherwise subthreshold doses (113).

Importantly, multisystem VACTERL (vertebral, anal, cardiac, tracheo-esophageal, renal, and limb) disorder has also been shown to be more common across Europe and has been formally causally related to cannabis exposure (201). As noted, this is a multisystem disease, and sonic hedgehog interference has been implicated in its pathoaetiology (357–359). Since cannabis is known to interfere with sonic hedgehog signaling both directly (111, 114–117, 346–355) and epigenomically (11), this further implicates cannabis in the teratology of these seven systems.

It has also been noted that teratologic syndromes otherwise uncharacterized have arisen across space and time in a manner causally related to cannabis exposure in Europe across the same period (201).

Importantly, the effects of alcohol have been shown to be mediated in part by the endocannabinoid system and associated epigenomic changes to the DNA methylation, histone structure, and chromatin architecture (360, 361). This implicates cannabinoids in the full spectrum of fetal alcohol spectrum disorders (FASD) in adults and young adults, in addition to their increasingly recognized role in developmental and congenital disruptions (360, 361).

#### 3.4.2. Daily cannabis use

Since much of the evidence points to high-dose cannabis use as being of utmost concern, it is of interest to quantify and define this key variable that is of the highest relevance to genotoxic and neurotoxic outcomes. As the best dataset for doing this is in the USA, the USA will be the nation of interest.

**Figure 1:**
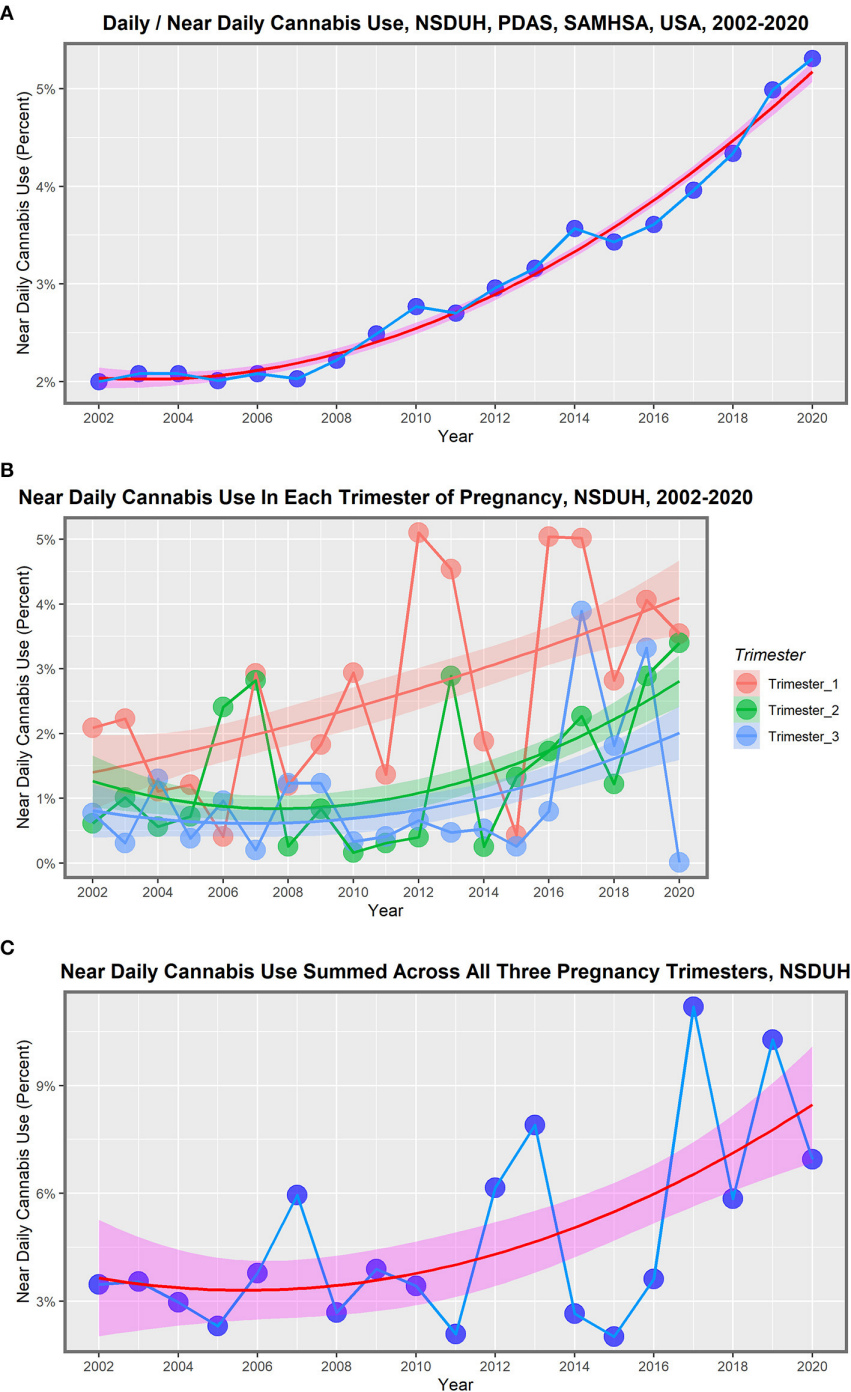
Daily cannabis use, the USA, 2002–2020. **(A)** Near daily cannabis use as reported at the national levels by NSDUH annual PDAS. **(B)** Near daily cannabis use in each of the trimesters of pregnancy by NSDUH. **(C)** Near daily cannabis use summed across all three pregnancy trimesters.

The most recent data on national drug use rates in the USA is available from the National Survey of Drug Use and Health conducted annually by the Substance Abuse and Mental Health Services Administration (362). Accessing the Public Use Data Analysis System website^38^ and running the data input code MRJMDAYS allows one to study the rates of daily or near-daily cannabis use^39^ across the whole population of individuals older than 12 years on an annual basis. The rate of near-daily cannabis use across the entire adult US population rose, as shown in Table 2, Figure 1A. This indicates that the rate of growth of cannabis devotees who smoked almost daily rose 265.5% nationally during 2002–2020. It should also be pointed out that the largest group in the survey comprised those who did not use cannabis at all, which in 2020 was 88.5%. Figure 1B shows the rate of near-daily use in each of the pregnancy trimesters. Figure 1C shows the rate of daily cannabis use summed across the three pregnancy trimesters.

**Table 2:** Daily cannabis use in the entire community and by pregnancy trimesters.

**Year**	**Near-daily use**	**First trimester pregnancy**	**Second trimester pregnancy**	**Third trimester pregnancy**	**Total pregnancy–summed**
2002	2.00%	2.09%	0.61%	0.77%	3.47%
2003	2.08%	2.23%	1.01%	0.31%	3.55%
2004	2.08%	1.11%	0.56%	1.30%	2.97%
2005	2.01%	1.21%	0.72%	0.38%	2.31%
2006	2.08%	0.41%	2.41%	0.96%	3.78%
2007	2.03%	2.93%	2.82%	0.20%	5.95%
2008	2.22%	1.20%	0.26%	1.23%	2.69%
2009	2.49%	1.83%	0.84%	1.23%	3.90%
2010	2.77%	2.94%	0.16%	0.33%	3.43%
2011	2.70%	1.37%	0.31%	0.41%	2.09%
2012	2.96%	5.10%	0.40%	0.66%	6.16%
2013	3.16%	4.54%	2.89%	0.47%	7.90%
2014	3.57%	1.88%	0.25%	0.53%	2.66%
2015	3.43%	0.43%	1.33%	0.26%	2.02%
2016	3.61%	5.04%	1.73%	0.80%	3.62%
2017	3.96%	5.02%	2.27%	3.89%	11.18%
2018	4.34%	2.82%	1.22%	1.81%	5.85%
2019	4.99%	4.06%	2.89%	3.32%	10.27%
2020	5.31%	3.54%	3.40%	0.01%	6.95%

**Figure 2:**
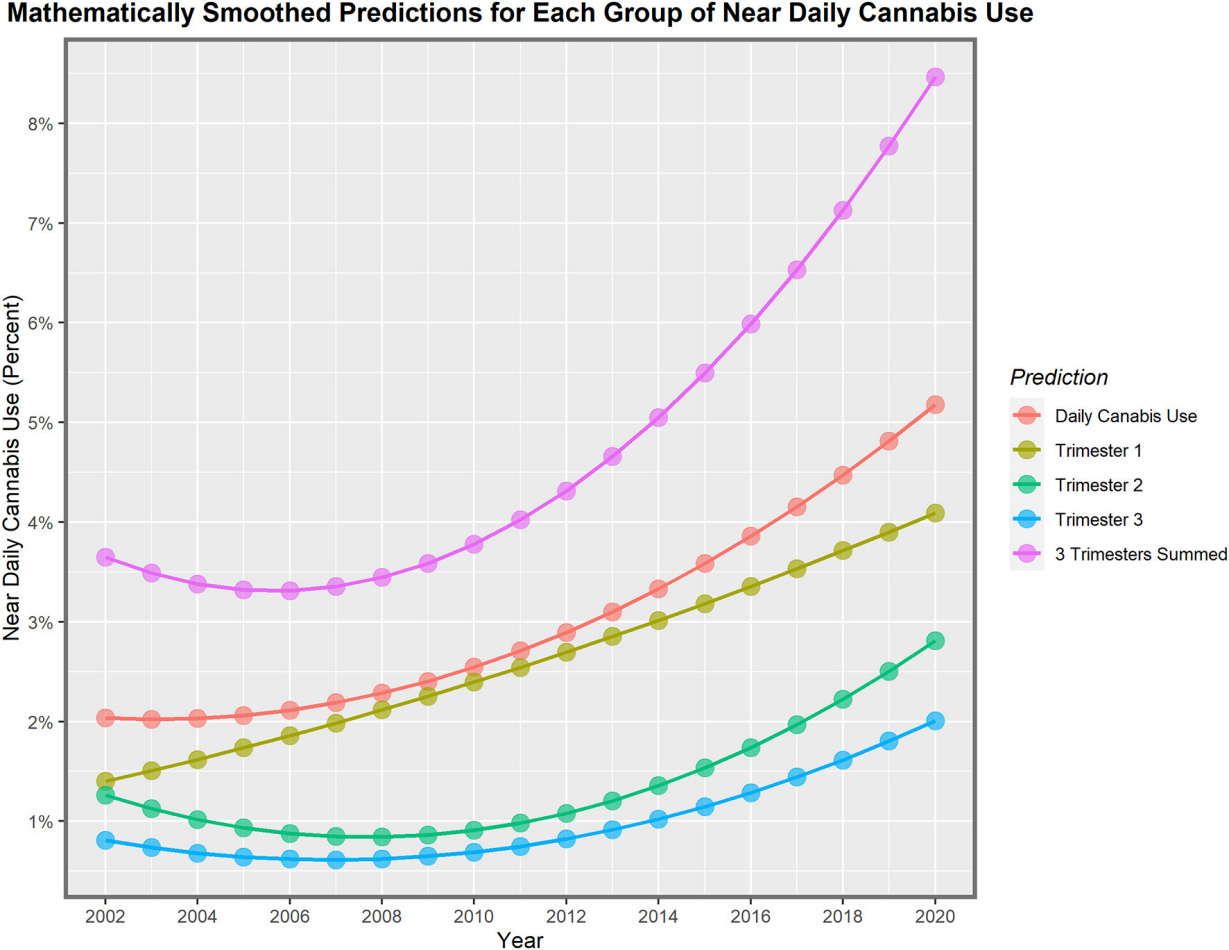
Modeled daily cannabis use.

These lines show a high degree of year-on-year variation. If one uses simple mathematical smoothing on these data and the quadratic polynomial, which is the most appropriate of the common models in the predict function in R, the data presented in Table 3 for smoothed modeled values is derived. If one compares the first period 2002–2010 to the second decade 2010–2020, the rise in the rate is clear in all cases. Daily cannabis use rose 24.9% in the first trimester and then 103.4% in the second trimester. The use in the second- and third-trimester use rose from −22.1% to 209.4% and −14.6% to 191.0%, respectively. The sum across all three pregnancy trimesters rose from 3.5% in the first period to 124.1% in the second period. Hence, these data demonstrate a greater rise across the board nationwide in near-daily cannabis use in all metrics and trimesters in the second decade. The first trimester is the only exception, where the relationship showed a linear modeled response across the whole period. These lines are all graphed in Figure 2.

**Table 3:** Modeled daily cannabis use (smoothed data).

**Year**	**Daily Cannabis Use**	**Trimester 1**	**Trimester 2**	**Trimester 3**	**All trimesters summed**
2002	0.02037	0.01400	0.01263	0.00808	0.03650
2003	0.02023	0.01507	0.01128	0.00736	0.03490
2004	0.02031	0.01619	0.01018	0.00681	0.03381
2005	0.02062	0.01736	0.00935	0.00642	0.03321
2006	0.02114	0.01858	0.00877	0.00619	0.03312
2007	0.02189	0.01985	0.00846	0.00612	0.03353
2008	0.02285	0.02117	0.00841	0.00622	0.03445
2009	0.02404	0.02254	0.00862	0.00648	0.03587
2010	0.02546	0.02396	0.00909	0.00690	0.03779
2011	0.02709	0.02543	0.00982	0.00749	0.04022
2012	0.02894	0.02695	0.01081	0.00824	0.04314
2013	0.03102	0.02852	0.01206	0.00915	0.04658
2014	0.03332	0.03014	0.01357	0.01022	0.05051
2015	0.03584	0.03182	0.01534	0.01146	0.05495
2016	0.03858	0.03354	0.01737	0.01286	0.05989
2017	0.04155	0.03531	0.01967	0.01442	0.06533
2018	0.04473	0.03714	0.02222	0.01615	0.07128
2019	0.04814	0.03901	0.02504	0.01804	0.07773
2020	0.05177	0.04094	0.02811	0.02009	0.08468
Interval	**Rise**	**Rise**	**Rise**	**Rise**	**Rise**
2002–2010	1.249	1.711	0.719	0.854	1.035
2010–2020	2.034	1.709	3.094	2.910	2.241

### 3.5. Epitranscriptomic metabolomics

RNA is subject to over 200 post-transcriptional modifications. The most common of these is m^6^ adenosine methylation (m^6^A). It has been shown that the m^6^A modification is applied to METTL16 ^40^ uniquely in leukaemic stem cells (LSCs), which creates a particular therapeutic vulnerability of LSCs. METTL16 deposits an m^6^A mark on the first and second codons for branched-chain amino acids (BCAA, including valine, leucine, and isoleucine) transaminases (BCAT1/2), which stabilizes the BCAA mRNAs and establishes them as a fundamental metabolic fuel for LSCs (363). Thus, the pathway is the METTL16/m^6^A/BCAT1/2/BCAA axis. Cancer-associated metabolic reprogramming has been shown to profoundly affect gene expression, differentiation, and tumor progression and is an emerging hallmark of malignancy. BCAT1/2 upregulation has been shown to be a marker of tumor aggressiveness across many tumor types. BCAAs are requisite to protein synthesis; they replenish TCA ^41^ intermediates and act as a nitrogen source for nucleotide synthesis via the glutamine–glutamate pathway. Therefore, the upregulation of BCAAs metabolically reprograms oxidative phosphorylation, the citric acid cycle, and nucleotide synthesis to fuel the rapid growth of malignant cells. AML cells are known to be addicted to BCAAs. METTL16 inhibition has been shown to drop LSC frequency 10-200-fold (363).

Some researchers worked with a standard model of acute myeloid leukemia and found that the mRNA for IGF2BP2^42^ is an m^6^A reader. This m^6^A reader stabilized the m^6^A modification of PRMT6^43^, which post-translationally modified histone H3R2me2a^44^. This modification suppressed the lipid transporter MFSD2A^45^, thereby reducing the lipid transport into LSCs (364). Indeed, approximately 60% of m^6^A targets were only observed in LSCs. It was also noted that m^6^A mRNA targets are enriched in immune checkpoint targets, which might be a key explanation of how LSCs avoid or subvert immunosurveillance (364).

## 4. Conclusion

The above considerations clearly demonstrate the salience and centrality of the epigenome, including the three-dimensional architecture of the nucleus, for determining gene expression and its major perturbation by cannabis exposure. Well-documented rising rates of daily cannabis use, cannabis use in pregnancy, and the currently amply demonstrated exponential cannabis genotoxic dose-response relationship imply that such studies are of primary importance and are a major research priority for addiction medicine, neuropsychiatric understandings, and public health management. These issues are pursued further in Part 2, which examines the metabolic and immunomic underpinning of these features and the manner in which these issues apply to neuronal toxicity and epigenotoxicity, along with the disruption of key events at the synapse. Specifically, these investigations elegantly demonstrate the importance and relevance of all of the considered levels of cellular machinery dysregulation.

## Author contributions

AR conceived the idea, performed the literature review, and wrote the first draft. GH added meaningful intellectual input, edited the first draft, provided project supervision and support, curated resources, and supervised the conduct of the project. All authors contributed to the article and approved the submitted version.

## Footnotes

^1^CB1R, Cannabinoid type 1 receptor.

^2^CB2R, Cannabinoid type 2 receptor.

^3^TRPV, Transient receptor potential voltage-gated channels.

^4^PPAR, Peroxisome proliferator activator receptor.

^5^GPCR, G-protein coupled receptor; GPR, G-protein receptor.

^6^NMDAR, N-methyl-D-aspartate receptor.

^7^GABA_A_R, γ-amino-butyric acid receptor.

^8^5HT_3_R, 5-hydroxytryptamine (serotonin) 3 receptor.

^9^CCL2, C-C motif chemokine ligand 2 (CCL2). Also known as monocyte chemoattractant protein 1 (MCP1).

^10^CCL20, C-C motif chemokine ligand 2 (CCL20). Also known as liver activation regulated chemokine (LARC) or macrophage inflammatory protein 3 (MIP3A).

^11^LINE1, Long interspersed nuclear elements 1.

^12^H3K27ac, Histone 3 lysine 27 acetylation.

^13^H3K4me3, Histone 3 lysine 4 trimethylation.

^14^Histone 3 lysine 9 trimethylation.

^15^Histone 3 lysine 27 trimethylation.

^16^CDKN1A, Cyclin-dependent kinase 1A.

^17^SIRT, Silencer of information transfer–a major sirtuin class silencing gene expression. Sirtuins also possess HDAC activity and together constitute a major class of HDACs.

^18^HDAC1, Histone deacetylase 1.

^19^PARP1, Poly (ADP-ribose) polymerase−1.

^20^CTCF, CCCTF-binding factor.

^21^SMC, Structural maintenance of chromosomes.

^22^RAD51, Recombinase/recombination protein A/RAD51 (*S. cerevisiae*) Homolog (*E coli* RecA homolog).

^23^STAG, Stromal antigen. STAG1 is also called SCC1. STAG2 is also called SCC3B. STAG3 is also called SCC3H3.

^24^GATA3, GATA binding Protein 3.

^25^SMARCA4, SWI/SNF-related, matrix-associated, actin-dependent regulator of chromatin, subfamily A, member 4.

^26^CRLF2, Cytokine receptor-like factor 2.

^27^JAK2, Janus kinase 2.

^28^STAT5, Signal transducer and activator of transcription 5.

^29^DNMT, DNA methyltransferases.

^30^TET, Ten-eleven translocation DNA methylcytosine dioxygenase.

^31^KMT, Lysine methyltransferase.

^32^KDM, Lysine demethylase.

^33^KAT, Lysine acetyl transferases.

^34^HDAC, Histone deacetylases.

^35^Sirtuins, Silencer of information transfer/histone/lysine deacetylases.

^36^SMARCA, SWI/SNF-related, matrix-associated, actin-dependent regulator of chromatin, subfamily A.

^37^UHRF1, Ubiquitin-like with PHD and ring finger domains 1.

^38^URL: https://pdas.samhsa.gov/#/ (accessed January 27^th^, 2023).

^39^Use of cannabis 20-30 days per month.

^40^METTL16, Methyltransferase 16, N6-methyladenosine.

^41^TCA, Tricarboxylic acid cycle, also known as citric acid cycle and Krebs cycle.

^42^IGF2BP2, Insulin-like growth factor 2 binding partner 2.

^43^PRMT, Protein arginine methyltransferase 6.

^44^H3R2me2a, Histone 3 arginine 2 demethylation.

^45^MFSD2A, MFSD2 lysolipid transporter A, lysophospholipi.
